# Supplementary material for: Blood glucose and subcutaneous continuous glucose monitoring in critically ill horses: A pilot study
Source: PLoS One. 2021 Feb 24;16(2):e0247561. doi: 10.1371/journal.pone.0247561 (PMC7904136; doi:10.1371/journal.pone.0247561)
Supplement: S1 Raw data set — (DOCX) [file pone.0247561.s001.docx]

**Difference between CMGS and POC/Acid base clinically acceptable or unacceptable?**

| **Case (Horses)** |  |
| --- | --- |
| **1** | \| **Clinically acceptable**  Difference between glucose value measurements being 1 mmol/L or lower. \| **CGMS vs POC:** 12/19 = 63,16%  **CGMS vs Acid Base:** 11/19 = 57,89% \| \| --- \| --- \| \| **Clinically unacceptable**  Difference between glucose value measurements being more than 1 mmol/L. \| **CGMS vs POC:** 7/19 = 36,84%  **CGMS vs Acid Base:** 8/19 = 42,11% \| |
| **2** | \| **Clinically acceptable**  Difference between glucose value measurements being 1 mmol/L or lower. \| **CGMS vs POC:** 6/9 = 66,67%  **CGMS vs Acid Base:** 7/9 = 77,78% \| \| --- \| --- \| \| **Clinically unacceptable**  Difference between glucose value measurements being more than 1 mmol/L. \| **CGMS vs POC:** 3/9 = 33,33%  **CGMS vs Acid Base:** 2/9 = 22,22% \| |
| **3** | \| **Clinically acceptable**  Difference between glucose value measurements being 1 mmol/L or lower. \| **CGMS vs POC:** 6/11 = 54,54%  **CGMS vs Acid Base:** 7/11 = 63,63% \| \| --- \| --- \| \| **Clinically unacceptable**  Difference between glucose value measurements being more than 1 mmol/L. \| **CGMS vs POC:** 5/11 = 45,45%  **CGMS vs Acid Base:** 4/11 = 36,36% \| |
| **6** | x |
| **8** | \| **Clinically acceptable**  Difference between glucose value measurements being 1 mmol/L or lower. \| **CGMS vs POC:** 6/14 = 42,86%  **CGMS vs Acid Base:** 5/14 = 35,71% \| \| --- \| --- \| \| **Clinically unacceptable**  Difference between glucose value measurements being more than 1 mmol/L. \| **CGMS vs POC:** 8/14 = 57,14%  **CGMS vs Acid Base:** 9/14 = 64,29% \| |

| **Cases (Foals)** |  |
| --- | --- |
| **4** | \| **Clinically acceptable**  Difference between glucose value measurements being 1 mmol/L or lower. \| **CGMS vs POC:** 9/11 = 81,81%  **CGMS vs Acid Base:** 10/11 = 90,9% \| \| --- \| --- \| \| **Clinically unacceptable**  Difference between glucose value measurements being more than 1 mmol/L. \| **CGMS vs POC:** 2/11 = 18,18%  **CGMS vs Acid Base:** 1/11 = 9,09% \| |
| **5** | x |
| **7** | x |
| **9** | x |
