# Supplementary material for: Blood glucose and subcutaneous continuous glucose monitoring in critically ill horses: A pilot study
Source: PLoS One. 2021 Feb 24;16(2):e0247561. doi: 10.1371/journal.pone.0247561 (PMC7904136; doi:10.1371/journal.pone.0247561)
Supplement: S2 Raw data set — (DOCX) [file pone.0247561.s002.docx]

| **Case (Horses)** |  |
| --- | --- |
| **1** | **CGMS vs POC:** POC has a tendency of measuring higher glucose value than CMGS (57,89%).  **CGMS vs Acid Base:** Acid Base has a tendency of measuring higher glucose value than CGMS (63,16%).  **POC vs Acid Base:** Acid Base has a tendency of measuring higher glucose value than POC (89,47%). |
| **2** | **CGMS vs POC:** CGMS has a tendency of measuring higher glucose value than POC (88,89%).  **CGMS vs Acid Base:** CGMS has a tendency of measuring higher glucose value than Acid Base (88,89%).  **POC vs Acid Base:** There is an equal amount of measurements measuring higher or lower glucose value. |
| **3** | **CGMS vs POC:** There is an equal amount of measurements measuring higher or lower glucose value.  **CGMS vs Acid Base:** CGMS has a tendency of measuring higher glucose value than Acid Base (63,63%).  **POC vs Acid Base:** POC has a tendency of measuring higher glucose value than Acid Base (72,72%) |
| **6** | x |
| **8** | **CGMS vs POC:**  POC has a tendency of measuring higher glucose value than CMGS (50%).  **CGMS vs Acid Base:** Acid Base has a tendency of measuring higher glucose value than CGMS (78,57%).  **POC vs Acid Base:**  Acid Base has a tendency of measuring higher glucose value than POC (85,57%). |

**Overview - Dataevaluation – Higher / lower**

| **Case (Foals)** |  |
| --- | --- |
| **4** | **CGMS vs POC:** POC has a tendency of measuring higher glucose value than CMGS (63,63%).  **CGMS vs Acid Base:** CGMS has a tendency of measuring higher glucose value than Acid Base (63,63%).  **POC vs Acid Base:** POC has a tendency of measuring higher glucose value than Acid Base (81,81%) |
| **5** | x |
| **7** | x |
| **9** | x |
