# Supplementary material for: Blood glucose and subcutaneous continuous glucose monitoring in critically ill horses: A pilot study
Source: PLoS One. 2021 Feb 24;16(2):e0247561. doi: 10.1371/journal.pone.0247561 (PMC7904136; doi:10.1371/journal.pone.0247561)
Supplement: S3 Raw data set — (DOCX) [file pone.0247561.s003.docx]

## Overview of % Normo-, hypo-, hyperglycemia

**Adult horses:
Normoglycemia (4,4 – 9 mmol/L)** (Normo)
**Hypoglycemia ( < 4,4 mmol/L)** (Hypo)
**Hyperglycemia (> 9 mmol/L)** (Hyper)

**Neonatal foals:
Normoglycemia (4,4 – 7 mmol/L)** (Normo)
**Hypoglycemia ( < 4,4 mmol/L)** (Hypo)
**Hyperglycemia (> 7 mmol/L)** (Hyper)

Time 0H with CGMS is not taken in calculation due to it is the same value as from POC 0H value

## Case 1 – Adult horse

| **Time (Hours)** | **CGMS** | **Blood gas** | **POC** |
| --- | --- | --- | --- |
| **0 H** | Normo | Normo | Normo |
| **4 H** | Hypo | Normo | Normo |
| **8 H** | Hypo | Hypo | Hypo |
| **12 H** | Hypo | Normo | Normo |
| **16 H** | Normo | Normo | Normo |
| **20 H** | Normo | Normo | Normo |
| **24 H** | Normo | Normo | Normo |
| **28 H** | Normo | Normo | Normo |
| **32 H** | Normo | Normo | Hypo |
| **36 H** | Normo | Normo | Normo |
| **40 H** | Normo | Normo | Normo |
| **44 H** | Normo | Normo | Normo |
| **48 H** | Normo | Normo | Hypo |
| **52 H** | Normo | Normo | Normo |
| **56 H** | Normo | Normo | Normo |
| **60 H** | Normo | Normo | Hyper |
| **64 H** | Normo | Normo | Normo |
| **68 H** | Normo | Hyper | Normo |
| **72 H** | Normo | Normo | Normo |
| **Summary:** | **Normo:** 15/18 = 83,33 %  **Hypo:** 3/18 = 16,67%  **Hyper:** 0% | **Normo:** 17/19 = 89,47%  **Hypo:** 1/19 = 5,26%  **Hyper:** 1/19 = 5,26% | **Normo:** 15/19 = 78,95%  **Hypo:** 3/19 = 15,79%  **Hyper:** 1/19 = 5,26% |

## Case 2 – Adult horse

| **Time (Hours)** | **CGMS** | **Blood gas** | **POC** |
| --- | --- | --- | --- |
| **0 H** | Hyper | Hyper | Hyper |
| **4 H** | Normo | Normo | Normo |
| **8 H** | Normo | Normo | Normo |
| **12 H** | Normo | Normo | Normo |
| **16 H** | Normo | Normo | Normo |
| **20 H** | Normo | Normo | Normo |
| **24 H** | Normo | Normo | Normo |
| **28 H** | Normo | Normo | Normo |
| **32 H** | Normo | Normo | Normo |
| **Summary:** | **Normo:** 8/8 = 100%  **Hypo:** 0%  **Hyper:** 0% | **Normo:** 8/9 = 88,89%  **Hypo:** 0%  **Hyper:** 1/9 = 11,11% | **Normo:** 8/9 = 88,89%  **Hypo:** 0%  **Hyper:** 1/9 = 11,11% |

## Case 3 – Adult horse

| **Time (Hours)** | **CGMS** | **Blood gas** | **POC** |
| --- | --- | --- | --- |
| **0 H** | Hyper | Hyper | Hyper |
| **4 H** | Hyper | Hyper | Hyper |
| **8 H** | Normo | Normo | Normo |
| **12 H** | Normo | Normo | Normo |
| **16 H** | Normo | Normo | Normo |
| **20 H** | Hyper | Hyper | Hyper |
| **24 H** | Hyper | Normo | Normo |
| **28 H** | Hyper | Normo | Hyper |
| **32 H** | Hyper | Hyper | Hyper |
| **36 H** | Normo | Normo | Normo |
| **40 H** | Normo | Normo | Normo |
| **Summary:** | **Normo:** 5/10 = 50%  **Hypo:** 0%  **Hyper:** 5/10 = 50% | **Normo:** 7/11 = 63,63%  **Hypo:** 0%  **Hyper:** 4/11 = 36,36% | **Normo:** 6/11 = 54,54%  **Hypo:** 0%  **Hyper:**  5/11 = 45,45% |

## Case 4 – Neonatal foal

| **Time (Hours)** | **CGMS** | **Blood gas** | **POC** |
| --- | --- | --- | --- |
| **0 H** | Normo | Normo | Normo |
| **4 H** | Normo | Normo | Normo |
| **8 H** | Normo | Normo | Normo |
| **12 H** | Normo | Normo | Normo |
| **16 H** | Normo | Normo | Normo |
| **20 H** | Normo | Normo | Normo |
| **24 H** | Normo | Normo | Normo |
| **28 H** | Normo | Normo | Normo |
| **32 H** | Normo | Normo | Hyper |
| **36 H** | Normo | Normo | Normo |
| **40 H** | Normo | Hyper | Hyper |
| **Summary:** | **Normo:** 100%  **Hypo:** 0%  **Hyper:** 0% | **Normo:** 10/11 = 90,9%  **Hypo:** 0%  **Hyper:** 1/11 = 9,09% | **Normo:** 2/11 = 18,18%  **Hypo:** 0%  **Hyper:** 9/11 = 81,81% |

## Case 5 – Neonatal foal

**Only one measurement**Blood gas (6,8 mmol/L): Normoglycemia
POC (7,2 mmol/L): Hyperglycemia

## Case 6 – Adult horse

**Only one measurement**Normoglycemia in Blood gas and POC

## Case 7 – Neonatal foal

**No measurement obtained**

## Case 8 – Adult horse

| **Time (Hours)** | **CGMS** | **Blood gas** | **POC** |
| --- | --- | --- | --- |
| **0 H** | Hyper | Hyper | Hyper |
| **4 H** | Hyper | Hyper | Hyper |
| **8 H** | Hyper | Hyper | Hyper |
| **12 H** | Normo | Normo | Normo |
| **16 H** | Normo | Normo | Normo |
| **20 H** | Normo | Normo | Normo |
| **24 H** | Normo | Normo | Normo |
| **28 H** | Hypo | Normo | Normo |
| **32 H** | Normo | Normo | Normo |
| **36 H** | Normo | Hyper | Normo |
| **40 H** | Hyper | Hyper | Hyper |
| **44 H** | Hyper | Hyper | Hyper |
| **48 H** | Normo | Hyper | Hyper |
| **52 H** | Hyper | Normo | Normo |
| **Summary:** | **Normo:** 7/13 = 53,85%  **Hypo:** 1/13 = 7,69%  **Hyper:** 5/13 = 38,46% | **Normo:** 7/14 = 50%  **Hypo:** 0%  **Hyper:** 7/14 = 50% | **Normo:** 8/14 = 57,14%  **Hypo:** 0%  **Hyper:** 6/14 = 42,86% |

## Case 9 – Neonatal foal

**Only one measurement**Blood gas (8,4 mmol/L): Hyperglycemia
POC (8,1 mmol/L): Hyperglycemia

## Case 10 – Adult horse

| **Time (Hours)** | **CGMS** | **Blood gas** | **POC** |
| --- | --- | --- | --- |
| **0 H** | Normo | Normo | Normo |
| **4 H** | Normo | Normo | Normo |
| **8 H** | Normo | Normo | Normo |
| **12 H** | Normo | Normo | Normo |
| **16 H** | Normo | Normo | Normo |
| **20 H** | Normo | Normo | Normo |
| **Summary:** | **Normo:** 100%  **Hypo:** 0%  **Hyper:** 0% | **Normo:** 6/6 = 100%  **Hypo:** 0%  **Hyper:** 0% | **Normo:** 100%  **Hypo:** 0%  **Hyper:** 0% |
