# Supplementary material for: Blood glucose and subcutaneous continuous glucose monitoring in critically ill horses: A pilot study
Source: PLoS One. 2021 Feb 24;16(2):e0247561. doi: 10.1371/journal.pone.0247561 (PMC7904136; doi:10.1371/journal.pone.0247561)
Supplement: S4 Raw data set — (DOCX) [file pone.0247561.s004.docx]

**Overview of cases from study start March the 1^st^ until April the 26^th^ 2016**

|  | **Number of CGMS sensors used** | **Time from placement of first sensor until CGMS monitoring starts (including initialization period of two hours )** | **Total time of glucose CGMS monitoring (hours after initialization period of two hours)** | **Benefits or disadvantages of CGMS over conventional monitoring** | **Statistical analysis** mean bias (95% limits of agreement) |
| --- | --- | --- | --- | --- | --- |
| **Case 1 (horse)** | Total number used: 5  Time from start of insertion to starting of data collection: 3  (1^st^ sensor: operator error; the horse moves, 2^nd^ sensor: monitor report: Sensor done, 3^rd^ sensor works)  (After 72 hours 2 sensors was tried to be placed; 4^th^ and 5^th^ sensor: operator error; the horse moves) | 5 hours | 71 hours and 30 minutes (~72 hours)  (after 71 hours and 30 minutes - report: sensor end) | **Benefits:**  🡪At 18:50 the 08.03.2016 the CGMS gives an alarm that the glucose is low. By a mistake the signal has been read as low signal?? There it has been 1 hour and 50 minutes from Acid base / Glucometer measurement and it is 2 hours and 10 minutes until next measurement. There it would have been possible to react and take a glucometer measurement for confirmation and even increase the IV glucose.  🡪At 02:20 the 10.03.2016 the CGMS gives an alarm that the glucose is high. There it has been 1 hour and 20 minutes since Acid base / Glucometer measurement and 2 hours and 40 minutes until next measurement. From 02:20 to 04:20 the CGMS measures glucose values from 9,1-11,9 mmol/L, then it becomes normoglycemia at 04:25. None of these high values are recorded with Acid base / Glucometer.  **Disadvantages:** 🡪5 hours from insertion of 1st sensor until data is collected.  🡪Because of inexperience? of inserting the sensor it means that too many sensors are put in wrongly.  🡪When the mare was getting livelier she got hard to hold still to insert the new sensor after 72 hours. Thus needing more than 2 persons to insert the sensor. In a busy hospital environment like at that time it can be hard to have more people to help inserting the sensor. | **CGMS vs. POC** 0.04 (-2,23 to 2,31) mmol/L  **CGMS vs. blood gas** -0.47 (-2,60 to 1,66) mmol/L **POC vs. blood gas** No normal distribution: 22% below to 6 % above |
| **Case 2 (horse)** | 3  (1st sensor: monitor report: Sensor end, 2nd sensor: lost signal, 3rd sensor works) | 5 hours | 32 hours and 30 minutes (~32 hours)  (after 32 hours and 30 minutes sensor is taken out in order to use the CGMS for case 3). | **Benefits:** None detected.  **Disadvantages: 🡪**The 3^rd^ sensor was the first one to work.  🡪5 hours from insertion of 1st sensor until data is collected. | **CGMS vs. POC** 0,69 (-0,56 to 1,94) mmol/L **CGMS vs. blood gas** 0,53 (-0,43 to 1,49)  **POC vs. blood gas** No normal distribution:12% below to 6 % above |
| **Case 3 (horse)** | 1 | 2 hours and 15 minutes | 39 hours and 50 minutes (~40 hours)  (after 39 hours and 50 minutes sensor is taken out because the horse was euthanized). | **Benefits:**  🡪Only one sensor used to get data for CGMS.  🡪Because of colic pain the gelding rolled few times and the sensor stayed in place.  🡪Insulin injection was given because at 09:50 the 14.04.2016 the alarm high glucose alarm went off on CGMS. The alarm was set to give notice when over 13 mmol/L. There had been taken Acid base / Glucometer measurement at 09:00 but the Acid base showed: 11,2 mmol/L and the Glucometer: 12,00 mmol/L. Because the CGMS was rising gradually it was decided at 11:00 to give insulin. This rise would not have been detected because next measurement would have been taken 3 hours and 10 minutes after the alarm went off.  🡪After that the high glucose alarm was set up to 14 mmol/L so that the monitor would not keep giving alarms. At 13:55 there was another alarm that said high glucose (14,0 mmol/L) and the clinic vet was noticed. Because of insulin injection at 11:00 nothing was done but neither Acid base nor Glucometer had detected this rise. At 13:00 the Acid base was 7,8 mmol/L and the Glucometer: 7,2 mmol/L.  **Disadvantages:** None detected. | **CGMS vs. POC** -0,15 (-3,67 to 3,38) mmol/  **CGMS vs. blood gas** 0,23 (-3,14 to 3,60) mmol/L  **POC vs. blood gas** 0,37(-0,65 to 1,39) mmol/L |
| **Case 4 (foal)** | 1 | 2 hours and 15 minutes | 40 hours and 10 minutes  (after 40 hours sensor is taken out, the foal is euthanized) | **Benefits:**  🡪Only one sensor used to get data for CGMS.  🡪The foal was handled a lot, switching sides every other hour and held up standing to suckle mare. The sensor stayed in place.  **Disadvantage:** None detected | **CGMS vs. POC** No normal distribution 33 % below to 73 % below  **CGMS vs. blood gas** No normal distribution: 27 % below to 36 % above **POC vs. blood gas** 0,37(-0,27 to 1,01) mmol/L |
| **Case 5 (foal)** | 4  (1^st^ + 2^nd^ sensor: operator error – the foal is very lively, 3^rd^ and 4^th^ sensor: lost signal) | 3 hours and 45 minutes | 50 minutes | **Benefits:**  None detected  **Disadvantages:**  🡪Many sensors used without success in getting good data from CGMS 🡪3 hours and 45 minutes from insertion of 1st sensor until data is collected. 🡪Only 50 minutes of CGMS data collected | **One measurement:** Glucometer measured 0,4 mmol/L higher than the acid base machine. |
| **Case 6 (horse)** | 3 (1^st^ and 2^nd^ sensor: lost signal, 3^rd^ sensor: Monitor report sensor error | 6 hours and 30 minutes | 1 hour and 35 minutes  (after 1 hours and 35 minutes the monitor report: sensor error) | **Benefits:**  None detected  **Disadvantages:**  🡪Many sensors used without success in getting good data from CGMS. 🡪6 hours and 30 minutes from insertion of 1st sensor until data is collected. 🡪Only 1 hour and 35 minutes of CGMS data collected. | **One measurement:**  Acid base and glucometer measured the same glucose value (8,3 mmol/L) |
| **Case 7 (foal)** | 4 (1^st^ sensor – insertion error because inserter jammed, 2^nd^ sensor put in manually - fell off, 3^rd^ and 4^th^ sensor manually inserted - no light on transmitter.) | 0 | 0 | **Benefits:** None detected.  **Disadvantages:** 🡪Many sensors used without success in getting any data from CGMS. | x |
| **Case 8 (horse)** | 1 | 2 hours and 5 minutes | 48 hours and 55 minutes | **Benefits:** 🡪Only one sensor used to get data for CGMS.  🡪It is possible to disconnect the transmitter from the sensor, leave the sensor in the patient, put a new sensor in another patient, get the system to work on that patient and when done reconnect with the old sensor again without problems.  🡪When putting in wrong calibration value, you have 15 minutes to put in a new one. In this case 6 minutes went by before the right calibration value was inserted and it did not affect the measurements after.  🡪At 09:16 the 06.05.2016, the alarm low glucose goes off. There it has been two hours since last acid base and glucometer measurement and there are two more hours until the next measurement is planned to be taken. The acid base had shown two hours before 6,1 mmol/L and the glucometer 4,5 mmol/L. Two hours later the acid base shows 7 mmol/L and the glucometer 5,9 mmol/L. This swing in glucose would not have been detected if not for CGMS and it would have been possible if necessary to react with for example giving more glucose.  **Disadvantages:**  🡪When reconnecting the transmitter to the old sensor it starts its initialization period again. | **CGMS vs. POC** -0,26 (-2,98 to 2,45) mmol/L  **CGMS vs. blood gas** -1,12 (-3,30 to 1,06) mmol/L  **POC vs. blood gas** -0,86(-2,02 to 0,31) mmol/L |
| **Case 9 (foal)** | 3 | 5 hours and 15 minutes | 1 hour and 45 minutes (2nd sensor) | **Benefits:** None detected  **Disadvantages:**  🡪Many sensors used without success in getting good data from CGMS.  🡪Foal doing a lot of spontaneous uncontrolled movements and the sensor starts to loosen and fall off.  🡪When handling the foal the sensor got pulled out. | **One measurement:**  Acid base measures 0,3 mmol/L higher than POC. |
| **Case 10 (pony)** | 1 | 2 hours and 12 minutes | 19 hours and 40 minutes | **Benefits:** 🡪Only one sensor used to get data for CGMS.  🡪Alarm of low glucose started twice in between blood sampling. (More details later). This value would not have been detected without CGMS.  **Disadvantages:**  🡪Fell easily off when horse scratched its head on the box door. | **CGMS vs. POC** -1,15(-4,12 to 1,82) mmol/L  **CGMS vs. blood gas** -1,42(-3,99 to 1,16) mmol/L  **POC vs. blood gas** -0,27 (-1,22 to 0,68) mmol/L |
| **Summary (All cases)** | 26 sensors | 33 hours and 52 minutes | 255 hours and 10 minutes |  |  |
| **Average per case** | 2,6 sensors | 3,09 hours | 25,51 hours |  |  |

**Maximum time of CGMS monitoring:** 71 hours and 30 minutes
**Minimum time of CGMS monitoring:** 50 minutes

**Maximum time from placement of first sensor until CGMS monitoring starts (including initialization period of two hours):** 6 hours and 30 minutes
**Minimum time from placement of first sensor until CGMS monitoring starts (including initialization period of two hours):** 2 hours and 5 minutes.

**Money**

Trial run: 2 sensors (414 dkk per sensor * 2) = 828 DKK
26 sensors in cases (414 dkk per sensor * 26) = 10.764 DKK
7 sensor left available at the hospital (414 dkk per sensor * 7) = 2.898 DKK

**Total amount of money used for sensors until** now (35 * 414): 14.490 DKK

For more details on money see Summary case 1-10 protocol 3 - accounting protocol
