# Supplementary material for: Blood glucose and subcutaneous continuous glucose monitoring in critically ill horses: A pilot study
Source: PLoS One. 2021 Feb 24;16(2):e0247561. doi: 10.1371/journal.pone.0247561 (PMC7904136; doi:10.1371/journal.pone.0247561)
Supplement: S5 Raw data set — (DOCX) [file pone.0247561.s005.docx]

**Overview of sensors**

| **Case (Horses)** | **Sensors – Possible failures** |
| --- | --- |
| **1** | 1^st^ sensor: Horse moves so the sensor did not stay  2^nd^ sensor: Wrong placement, probably too ventrally placed or blood in the stylet.  3^rd^ sensor: Worked for 71 hours and 30 minutes  4^th^ sensor: Horse moves so the sensor did not stay  5^th^ sensor: Horse moves so much the sensor did not stay. |
| **2** | 1^st^ sensor: Malplaced – too ventrally  2^nd^ sensor: Probably fell out after an hour. Could have been movement of the horse or all the tape/tenso pulling.  3^rd^ sensor: Worked for 32 hours and 30 minutes before taken out to put in next case. Could probably work for a longer period. |
| **3** | 1^st^ sensor: Worked for 39 hours and 50 minutes before taken out because of euthanasia. Could probably work for a longer period. |
| **6** | 1^st^ sensor: Placed manually. Looked fine, could be blood in stylet, or maybe a functional error in the sensor? Keeps getting signal loss.  2^nd^ sensor: Looks like it has been pulled out from the patient – possibly by the tape and tenso  Before insertion of 3^rd^ sensor a test of the system was made and everything worked.  3^rd^ sensor: Worked for 1 hour and 35 minutes. Monitor reports sensor error. Possible that the sensor has fallen out or got clotted? |
| **8** | 1^st^ sensor: Worked for 48 hours and 55 minutes before taken out because of euthanasia of the patient. Could probably work for a longer period. |
| **10** | 1^st^ sensor: Worked for approx. 20 hours before pulled out by the pony. |

| **Cases (Foals)** | **Sensors – Possible failures** |
| --- | --- |
| **4** | 1^st^ sensor: Worked for 40 hours before taken out because of euthanasia of the patient. Could probably work for a longer period. |
| **5** | 1^st^ sensor: Inserted with inserter while foal was standing and held by a helper. No success with inserter because it is too big and foal was lively.  2^nd^ sensor: Inserted manually while foal was standing and held by a helper. No success because the foal was very lively.  3^rd^ sensor: Inserted manually while foal was standing and held by a helper. Insertion was a success but it was not able to get connection between sensor and monitor. Possible insertion error or mal-placement.  4^th^ sensor: Inserted manually while foal was lying because it was tired. Success with insertion and connection between sensor and monitor. After 50 minutes of data then alarm: signal loss. Possible that the sensor had fallen out because the foal was very active and looking towards the abdomen all the time. |
| **7** | 1^st^ sensor: Inserted with inserter and failed. The inserter jammed, thus not being able to insert the sensor on the skin.  2^nd^ sensor: Manual insertion. Was sitting good but then the foal moved its head and neck a lot so that the sensor fell out.  3^rd^ sensor: Manual insertion. Sitting good except for the glue on the sensor was hard to take away when gluing. When connecting the transmitter on sensor there was no light at connection  4^th^ sensor: Manual insertion. Sensor was well placed but when connecting the green light on the transmitter did not flash when connecting to the sensor. Possible that sensor was mal-placed or fallen out. |
| **9** | 1^st^ sensor: Sitting nicely. Alarm: Sensor week signal. 1st calibration moved from 22:30 to 00:46. It is likely that the transmitter and sensor had lost contact because when touching the transmitter it started to blink green light again. Fell out before initialization period was finished. The foal was moving a lot with very spontaneous and uncontrollable movements.  2^nd^ sensor: Worked for 1 hour and 45 minutes. The sensor was pulled out (properly by the foal/people handling the foal)  3^rd^ sensor: Sitting nicely. The sensor was accidently pulled out by people handling the foal before initialization period was finished. |
