# Supplementary material for: Blood glucose and subcutaneous continuous glucose monitoring in critically ill horses: A pilot study
Source: PLoS One. 2021 Feb 24;16(2):e0247561. doi: 10.1371/journal.pone.0247561 (PMC7904136; doi:10.1371/journal.pone.0247561)
Supplement: S6 Raw data set — (DOCX) [file pone.0247561.s006.docx]

# Pooled data for Adult horses (Case 1,2,3,8,10)

## With 0H

> CGMS <- c(5.8, 4.1, 3.4, 3.9, 5.1, 6.9, 6.5, 6.9, 5.3, 7.7, 5.8, 5.8, 5.7, 6.8, 8.8, 8.1, 6.5, 8.2, 4.9, 13.2, 7.9, 7.0, 6.4, 7.2, 6.3, 8.1, 6.7, 7.4, 11.8, 10.0, 7.0, 5.0, 4.8, 10.2, 9.9, 11.7, 10.8, 8.7, 7.7, 11.6, 10, 10.7, 6.8, 6.3, 4.8, 5.9, 4.3, 6.8, 7.5, 11.7, 9.1, 8, 9.1, 7.1, 4.8, 5.8, 5.4, 6.1, 7.7)

> Bloodgas <- c(6.3, 5.2, 3.9, 5.7, 7.8, 8.3, 7.6, 7.7, 4.9, 6.4, 5.2, 5.2, 5.0, 7.5, 8.2, 8.3, 5.7, 9.8, 6.4, 12.3, 6.6, 6.6, 6.5, 6.9, 5.6, 7.0, 6.6, 7.3, 10.8, 9.3, 6.6, 5.5, 8.0, 11.6, 7.8, 8.4, 11.4, 8.1, 7.6, 12.6, 10.5, 11.7, 6.4, 6.1, 7, 7.8, 5.8, 8, 9.3, 14.5, 11.1, 9.6, 7.9, 7.6, 8.5, 7.7, 5.8, 7.8, 8.0)

> POC <- c(5.8, 4.4, 3.6, 4.8, 7.4, 7.8, 6.8, 7.6, 4.1, 5.4, 5.2, 4.5, 4.1, 7.3, 7.9, 9.2, 5.1, 9.0, 5.5, 13.2, 6.1, 6.1, 6.2, 6.8, 5.2, 6.7, 6.4, 7.3, 11.8, 9.7, 6.6, 5.3, 8.3, 12.0, 7.2, 9.2, 12.6, 8.6, 7.9, 11.6, 9.6, 9.9, 4.9, 4.5, 5.9, 7.3, 4.7, 6.8, 8.5, 14.6, 10.7, 9.3, 8, 7.1, 8.2, 7.7, 4.7, 8.0, 8.1)

**POC vs Blood gas pooling (inkluderet case 6)**

> Bloodgas <- c(6.3, 5.2, 3.9, 5.7, 7.8, 8.3, 7.6, 7.7, 4.9, 6.4, 5.2, 5.2, 5.0, 7.5, 8.2, 8.3, 5.7, 9.8, 6.4, 12.3, 6.6, 6.6, 6.5, 6.9, 5.6, 7.0, 6.6, 7.3, 10.8, 9.3, 6.6, 5.5, 8.0, 11.6, 7.8, 8.4, 11.4, 8.1, 7.6, 12.6, 10.5, 11.7, 6.4, 6.1, 7, 7.8, 5.8, 8, 9.3, 14.5, 11.1, 9.6, 7.9, 7.6, 8.5, 7.7, 5.8, 7.8, 8.0, 8.3)

> POC <- c(5.8, 4.4, 3.6, 4.8, 7.4, 7.8, 6.8, 7.6, 4.1, 5.4, 5.2, 4.5, 4.1, 7.3, 7.9, 9.2, 5.1, 9.0, 5.5, 13.2, 6.1, 6.1, 6.2, 6.8, 5.2, 6.7, 6.4, 7.3, 11.8, 9.7, 6.6, 5.3, 8.3, 12.0, 7.2, 9.2, 12.6, 8.6, 7.9, 11.6, 9.6, 9.9, 4.9, 4.5, 5.9, 7.3, 4.7, 6.8, 8.5, 14.6, 10.7, 9.3, 8, 7.1, 8.2, 7.7, 4.7, 8.0, 8.1, 8.3)

## Uden 0H

> CGMS <- c(4.1, 3.4, 3.9, 5.1, 6.9, 6.5, 6.9, 5.3, 7.7, 5.8, 5.8, 5.7, 6.8, 8.8, 8.1, 6.5, 8.2, 4.9, 7.9, 7.0, 6.4, 7.2, 6.3, 8.1, 6.7, 7.4, 10.0, 7.0, 5.0, 4.8, 10.2, 9.9, 11.7, 10.8, 8.7, 7.7, 10, 10.7, 6.8, 6.3, 4.8, 5.9, 4.3, 7.5, 11.7, 9.1, 8, 9.1, 4.8, 5.8, 5.4, 6.1, 7.7)

> Bloodgas <- c(5.2, 3.9, 5.7, 7.8, 8.3, 7.6, 7.7, 4.9, 6.4, 5.2, 5.2, 5.0, 7.5, 8.2, 8.3, 5.7, 9.8, 6.4, 6.6, 6.6, 6.5, 6.9, 5.6, 7.0, 6.6, 7.3, 9.3, 6.6, 5.5, 8.0, 11.6, 7.8, 8.4, 11.4, 8.1, 7.6, 10.5, 11.7, 6.4, 6.1, 7, 7.8, 5.8, 9.3, 14.5, 11.1, 9.6, 7.9, 8.5, 7.7, 5.8, 7.8, 8.0)

> POC<- c(4.4, 3.6, 4.8, 7.4, 7.8, 6.8, 7.6, 4.1, 5.4, 5.2, 4.5, 4.1, 7.3, 7.9, 9.2, 5.1, 9.0, 5.5, 6.1, 6.1, 6.2, 6.8, 5.2, 6.7, 6.4, 7.3, 9.7, 6.6, 5.3, 8.3, 12.0, 7.2, 9.2, 12.6, 8.6, 7.9, 9.6, 9.9, 4.9, 4.5, 5.9, 7.3, 4.7, 8.5, 14.6, 10.7, 9.3, 8, 8.2, 7.7, 4.7, 8.0, 8.1)

**CGMS vs Blood gas – uden 0H**

**MedCalc**

| Method A | CGMS |
| --- | --- |
| Method B | Bloodgas |

| **Differences** |
| --- |

| Sample size | 53 |
| --- | --- |
| Arithmetic mean | -0.5170 |
| Standard deviation | 1.2382 |
| Lower limit | -2.9438 |
| Upper limit | 1.9099 |

**Bland Altman**

> bland.altman.plot(CGMS, Bloodgas, main="Adult horses - Bland Altman Plot CGMS vs Blood gas", xlab=" Average glucose concentration (mmol/L) by two assays ", ylab="Difference in glucose conc. (mmol/L) [CGMS-Blood gas]")


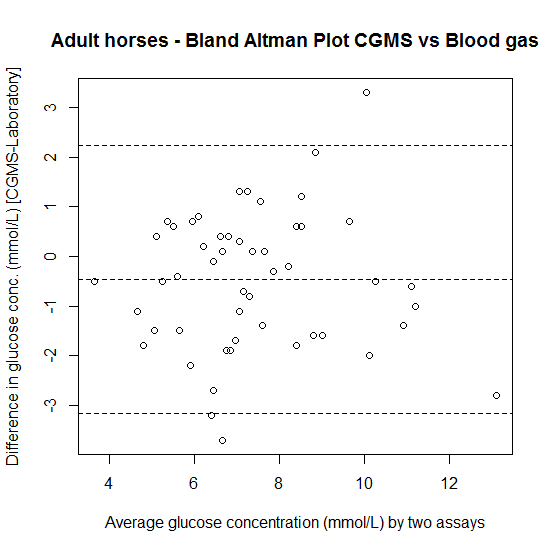


> bland.altman.plot(CGMS, Bloodgas, main="Adult horses - Bland Altman Plot CGMS vs Blood gas", xlab=" Average glucose concentration (mmol/L) by two assays", ylab=" Difference in glucose conc. (mmol/L) [CGMS-Blood gas]", conf.int=0.95, silent=FALSE)


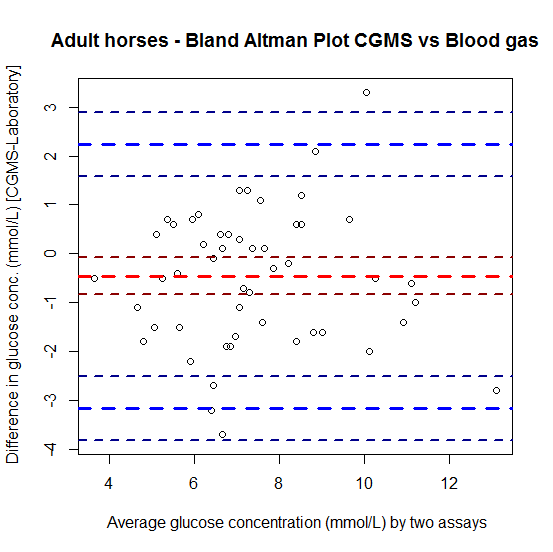


$means
 [1] 4.65 3.65 4.80 6.45 7.60 7.05 7.30 5.10 7.05 5.50 5.50 5.35
[13] 7.15 8.50 8.20 6.10 9.00 5.65 7.25 6.80 6.45 7.05 5.95 7.55
[25] 6.65 7.35 9.65 6.80 5.25 6.40 10.90 8.85 10.05 11.10 8.40 7.65
[37] 10.25 11.20 6.60 6.20 5.90 6.85 5.05 8.40 13.10 10.10 8.80 8.50
[49] 6.65 6.75 5.60 6.95 7.85

$diffs
 [1] -1.1 -0.5 -1.8 -2.7 -1.4 -1.1 -0.8 0.4 1.3 0.6 0.6 0.7 -0.7 0.6 -0.2 9
[16] 0.8 -1.6 -1.5 1.3 0.4 -0.1 0.3 0.7 1.1 0.1 0.1 0.7 0.4 -0.5 -3.2 19
[31] -1.4 2.1 3.3 -0.6 0.6 0.1 -0.5 -1.0 0.4 0.2 -2.2 -1.9 -1.5 -1.8 -2.8 26
[46] -2.0 -1.6 1.2 -3.7 -1.9 -0.4 -1.7 -0.3 28

$diffs
 [1] -1.1 -0.5 -1.8 -2.7 -1.4 -1.1 -0.8 0.4 1.3 0.6 0.6 0.7 -0.7 0.6 -0.2 9
[16] 0.8 -1.6 -1.5 1.3 0.4 -0.1 0.3 0.7 1.1 0.1 0.1 0.7 0.4 -0.5 -3.2 14
[31] -1.4 2.1 3.3 -0.6 0.6 0.1 -0.5 -1.0 0.4 0.2 -2.2 -1.9 -1.5 -1.8 -2.8 23
[46] -2.0 -1.6 1.2 -3.7 -1.9 -0.4 -1.7 -0.3 30

$groups
 group1 group2
1 4.1 5.2
2 3.4 3.9
3 3.9 5.7
4 5.1 7.8
5 6.9 8.3
6 6.5 7.6
7 6.9 7.7
8 5.3 4.9
9 7.7 6.4
10 5.8 5.2
11 5.8 5.2
12 5.7 5.0
13 6.8 7.5
14 8.8 8.2
15 8.1 8.3
16 6.5 5.7
17 8.2 9.8
18 4.9 6.4
19 7.9 6.6
20 7.0 6.6
21 6.4 6.5
22 7.2 6.9
23 6.3 5.6
24 8.1 7.0
25 6.7 6.6
26 7.4 7.3
27 10.0 9.3
28 7.0 6.6
29 5.0 5.5
30 4.8 8.0
31 10.2 11.6
32 9.9 7.8
33 11.7 8.4
34 10.8 11.4
35 8.7 8.1
36 7.7 7.6
37 10.0 10.5
38 10.7 11.7
39 6.8 6.4
40 6.3 6.1
41 4.8 7.0
42 5.9 7.8
43 4.3 5.8
44 7.5 9.3
45 11.7 14.5
46 9.1 11.1
47 8.0 9.6
48 9.1 7.9
49 4.8 8.5
50 5.8 7.7
51 5.4 5.8
52 6.1 7.8
53 7.7 8.0

$based.on
[1] 53

$lower.limit
[1] -3.157703

$mean.diffs
[1] -0.4622642

$upper.limit
[1] 2.233175

$lines
lower.limit mean.diffs upper.limit
 -3.1577031 -0.4622642 2.2331748

$CI.lines
lower.limit.ci.lower lower.limit.ci.upper mean.diff.ci.lower
 -3.81425198 -2.50115423 -0.84132282
mean.diff.ci.upper upper.limit.ci.lower upper.limit.ci.upper
 -0.08320548 1.57662593 2.88972367

$two
[1] 1.96

$critical.diff
[1] 2.695439

**Shapiro-Wilk normality test**

> shapiro.test(CGMS-Bloodgas)
W = 0.98277, p-value = 0.6373

- P-value over 0.05 🡪 the hypothesis that there is normal distribution on the difference between CGMS and Blood gas (Laboratory) is accepted.

**CGMS vs POC – uden 0H**

**MedCalc**

| Method A | CGMS |
| --- | --- |
| Method B | POC |

| **Differences** |
| --- |

| Sample size | 53 |
| --- | --- |
| Arithmetic mean | -0.1604 |
| Standard deviation | 1.3334 |
| Lower limit | -2.7738 |
| Upper limit | 2.4530 |

**BlandAltman**

> bland.altman.plot(CGMS, POC, main="Adult horses - Bland Altman Plot CGMS vs POC", xlab=" Average glucose concentration (mmol/L) by two assays", ylab="Difference in glucose conc. (mmol/L) [CGMS-POC]")


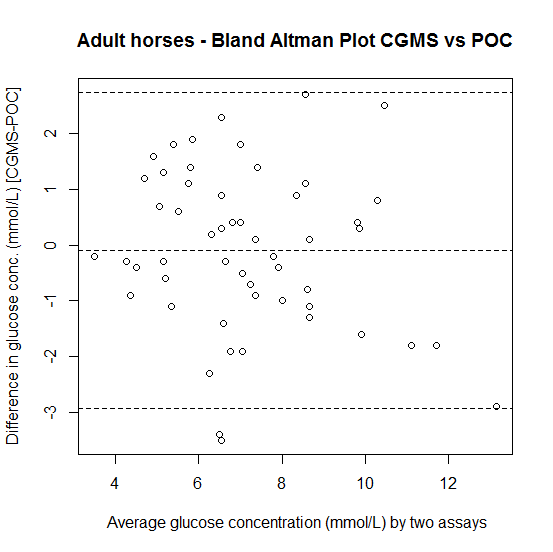


> bland.altman.plot(CGMS, POC, main="Adult horses - Bland Altman Plot CGMS vs POC", xlab="Average glucose concentration (mmol/L) by two assays", ylab="Difference in glucose conc. (mmol/L) [CGMS-POC]", conf.int=0.95, silent=FALSE)


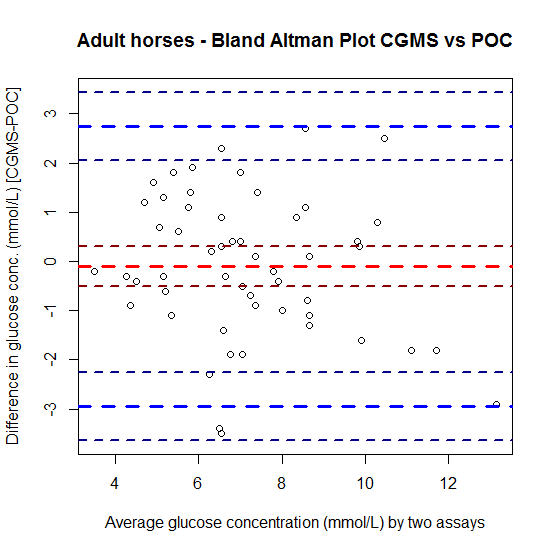


$means
 [1] 4.25 3.50 4.35 6.25 7.35 6.65 7.25 4.70 6.55 5.50 5.15 4.90
[13] 7.05 8.35 8.65 5.80 8.60 5.20 7.00 6.55 6.30 7.00 5.75 7.40
[25] 6.55 7.35 9.85 6.80 5.15 6.55 11.10 8.55 10.45 11.70 8.65 7.80
[37] 9.80 10.30 5.85 5.40 5.35 6.60 4.50 8.00 13.15 9.90 8.65 8.55
[49] 6.50 6.75 5.05 7.05 7.90

$diffs
 [1] -0.3 -0.2 -0.9 -2.3 -0.9 -0.3 -0.7 1.2 2.3 0.6 1.3 1.6 -0.5 0.9 -1.1 9
[16] 1.4 -0.8 -0.6 1.8 0.9 0.2 0.4 1.1 1.4 0.3 0.1 0.3 0.4 -0.3 -3.5 19
[31] -1.8 2.7 2.5 -1.8 0.1 -0.2 0.4 0.8 1.9 1.8 -1.1 -1.4 -0.4 -1.0 -2.9 25
[46] -1.6 -1.3 1.1 -3.4 -1.9 0.7 -1.9 -0.4 27

$diffs
 [1] -0.3 -0.2 -0.9 -2.3 -0.9 -0.3 -0.7 1.2 2.3 0.6 1.3 1.6 -0.5 0.9 -1.1 9
[16] 1.4 -0.8 -0.6 1.8 0.9 0.2 0.4 1.1 1.4 0.3 0.1 0.3 0.4 -0.3 -3.5 13
[31] -1.8 2.7 2.5 -1.8 0.1 -0.2 0.4 0.8 1.9 1.8 -1.1 -1.4 -0.4 -1.0 -2.9 21
[46] -1.6 -1.3 1.1 -3.4 -1.9 0.7 -1.9 -0.4 27

$groups
 group1 group2
1 4.1 4.4
2 3.4 3.6
3 3.9 4.8
4 5.1 7.4
5 6.9 7.8
6 6.5 6.8
7 6.9 7.6
8 5.3 4.1
9 7.7 5.4
10 5.8 5.2
11 5.8 4.5
12 5.7 4.1
13 6.8 7.3
14 8.8 7.9
15 8.1 9.2
16 6.5 5.1
17 8.2 9.0
18 4.9 5.5
19 7.9 6.1
20 7.0 6.1
21 6.4 6.2
22 7.2 6.8
23 6.3 5.2
24 8.1 6.7
25 6.7 6.4
26 7.4 7.3
27 10.0 9.7
28 7.0 6.6
29 5.0 5.3
30 4.8 8.3
31 10.2 12.0
32 9.9 7.2
33 11.7 9.2
34 10.8 12.6
35 8.7 8.6
36 7.7 7.9
37 10.0 9.6
38 10.7 9.9
39 6.8 4.9
40 6.3 4.5
41 4.8 5.9
42 5.9 7.3
43 4.3 4.7
44 7.5 8.5
45 11.7 14.6
46 9.1 10.7
47 8.0 9.3
48 9.1 8.0
49 4.8 8.2
50 5.8 7.7
51 5.4 4.7
52 6.1 8.0
53 7.7 8.1

$based.on
[1] 53

$lower.limit
[1] -2.939269

$mean.diffs
[1] -0.1

$upper.limit
[1] 2.739269

$lines
lower.limit mean.diffs upper.limit
 -2.939269 -0.100000 2.739269

$CI.lines
lower.limit.ci.lower lower.limit.ci.upper mean.diff.ci.lower
 -3.6308520 -2.2476865 -0.4992855
 mean.diff.ci.upper upper.limit.ci.lower upper.limit.ci.upper
 0.2992855 2.0476865 3.4308520

$two
[1] 1.96

$critical.diff
[1] 2.839269

**Shapiro-Wilk normality test**> shapiro.test(CGMS-POC)
W = 0.98628, p-value = 0.7995

- P-value over 0.05 🡪 the hypothesis that there is normal distribution on the difference between CGMS and Blood gas (Laboratory) is accepted.

**POC vs Bloodgas – With 0H and including Case 6!**

**MedCalc**

| Method A | POC |
| --- | --- |
| Method B | Bloodgas |

| **Differences** |
| --- |

| Sample size | 60 |
| --- | --- |
| Arithmetic mean | -0.3417 |
| Standard deviation | 0.6473 |
| Lower limit | -1.6103 |
| Upper limit | 0.9270 |

**BlandAltman**

> bland.altman.plot(POC, Bloodgas, main="Adult horses - Bland Altman Plot POC vs Blood gas", xlab="Average glucose concentration (mmol/L) by two assays", ylab="Difference in glucose conc. (mmol/L) [POC-Blood gas]")


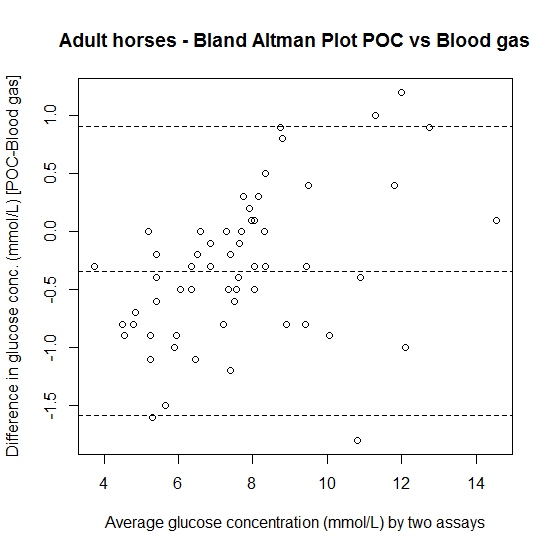


> bland.altman.plot(POC, Bloodgas, main="Adult horses - Bland Altman Plot POC vs Blood gas", xlab="Average glucose concentration (mmol/L) by two assays", ylab="Difference in glucose conc. (mmol/L) [POC-Blood gas]", conf.int=0.95, silent=FALSE)


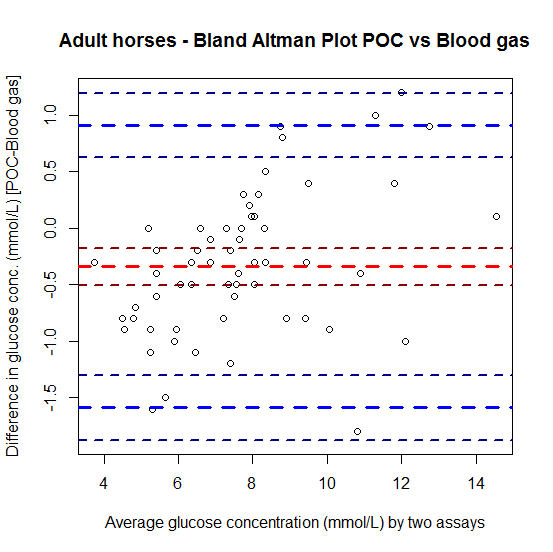


$means
 [1] 6.05 4.80 3.75 5.25 7.60 8.05 7.20 7.65 4.50 5.90 5.20 4.85
[13] 4.55 7.40 8.05 8.75 5.40 9.40 5.95 12.75 6.35 6.35 6.35 6.85
[25] 5.40 6.85 6.50 7.30 11.30 9.50 6.60 5.40 8.15 11.80 7.50 8.80
[37] 12.00 8.35 7.75 12.10 10.05 10.80 5.65 5.30 6.45 7.55 5.25 7.40
[49] 8.90 14.55 10.90 9.45 7.95 7.35 8.35 7.70 5.25 7.90 8.05 8.30

$diffs
 [1] -0.5 -0.8 -0.3 -0.9 -0.4 -0.5 -0.8 -0.1 -0.8 -1.0 0.0 -0.7 -0.9 -0.2 -0.3
[16] 0.9 -0.6 -0.8 -0.9 0.9 -0.5 -0.5 -0.3 -0.1 -0.4 -0.3 -0.2 0.0 1.0 0.4
[31] 0.0 -0.2 0.3 0.4 -0.6 0.8 1.2 0.5 0.3 -1.0 -0.9 -1.8 -1.5 -1.6 -1.1
[46] -0.5 -1.1 -1.2 -0.8 0.1 -0.4 -0.3 0.1 -0.5 -0.3 0.0 -1.1 0.2 0.1 0.0

$diffs
 [1] -0.5 -0.8 -0.3 -0.9 -0.4 -0.5 -0.8 -0.1 -0.8 -1.0 0.0 -0.7 -0.9 -0.2 -0.3
[16] 0.9 -0.6 -0.8 -0.9 0.9 -0.5 -0.5 -0.3 -0.1 -0.4 -0.3 -0.2 0.0 1.0 0.4
[31] 0.0 -0.2 0.3 0.4 -0.6 0.8 1.2 0.5 0.3 -1.0 -0.9 -1.8 -1.5 -1.6 -1.1
[46] -0.5 -1.1 -1.2 -0.8 0.1 -0.4 -0.3 0.1 -0.5 -0.3 0.0 -1.1 0.2 0.1 0.0

$groups
 group1 group2
1 5.8 6.3
2 4.4 5.2
3 3.6 3.9
4 4.8 5.7
5 7.4 7.8
6 7.8 8.3
7 6.8 7.6
8 7.6 7.7
9 4.1 4.9
10 5.4 6.4
11 5.2 5.2
12 4.5 5.2
13 4.1 5.0
14 7.3 7.5
15 7.9 8.2
16 9.2 8.3
17 5.1 5.7
18 9.0 9.8
19 5.5 6.4
20 13.2 12.3
21 6.1 6.6
22 6.1 6.6
23 6.2 6.5
24 6.8 6.9
25 5.2 5.6
26 6.7 7.0
27 6.4 6.6
28 7.3 7.3
29 11.8 10.8
30 9.7 9.3
31 6.6 6.6
32 5.3 5.5
33 8.3 8.0
34 12.0 11.6
35 7.2 7.8
36 9.2 8.4
37 12.6 11.4
38 8.6 8.1
39 7.9 7.6
40 11.6 12.6
41 9.6 10.5
42 9.9 11.7
43 4.9 6.4
44 4.5 6.1
45 5.9 7.0
46 7.3 7.8
47 4.7 5.8
48 6.8 8.0
49 8.5 9.3
50 14.6 14.5
51 10.7 11.1
52 9.3 9.6
53 8.0 7.9
54 7.1 7.6
55 8.2 8.5
56 7.7 7.7
57 4.7 5.8
58 8.0 7.8
59 8.1 8.0
60 8.3 8.3

$based.on
[1] 60

$lower.limit
[1] -1.590329

$mean.diffs
[1] -0.3416667

$upper.limit
[1] 0.9069958

$lines
lower.limit mean.diffs upper.limit
 -1.5903291 -0.3416667 0.9069958

$CI.lines
lower.limit.ci.lower lower.limit.ci.upper mean.diff.ci.lower
 -1.8753784 -1.3052797 -0.5062400
 mean.diff.ci.upper upper.limit.ci.lower upper.limit.ci.upper
 -0.1770933 0.6219464 1.1920451

$two
[1] 1.96

$critical.diff
[1] 1.248662

**Shapiro-Wilk normality test**> shapiro.test(POC-Bloodgas)
W = 0.98399, p-value = 0.6287

- P-value over 0.05 🡪 the hypothesis that there is normal distribution on the difference between CGMS and Blood gas (Laboratory) is accepted.
