# Supplementary material for: Blood glucose and subcutaneous continuous glucose monitoring in critically ill horses: A pilot study
Source: PLoS One. 2021 Feb 24;16(2):e0247561. doi: 10.1371/journal.pone.0247561 (PMC7904136; doi:10.1371/journal.pone.0247561)
Supplement: S7 Raw data set — (DOCX) [file pone.0247561.s007.docx]

**Case 1**

**Table xx. Case 1. Results of glucose measurements and physiological status.**

| Time (Hours) | CGMS  (mmol/L) | Blood gas  (mmol/L) | POC  (mmol/L) | Hematocrit (%) | Lactate (mmol/L) | pO_2_  (mmHg) | pH |
| --- | --- | --- | --- | --- | --- | --- | --- |
| 0 H | 5.8 | 6.3 | 5.8 | 43 | 0.9 | 33.2 | 7.345 |
| 4 H | 4.1 | 5.2 | 4.4 | 42 | 0.9 | 33.9 | 7.366 |
| 8 H | 3.4 | 3.9 | 3.6 | 41 | 0.8 | 45.8 | 7.344 |
| 12 H | 3.9 | 5.7 | 4.8 | 39 | 0.8 | 43.7 | 7.359 |
| 16 H | 5.1 | 7.8 | 7.4 | 38 | 0.7 | 38.0 | 7.384 |
| 20 H | 6.9 | 8.3 | 7.8 | 39 | 0.7 | 31.9 | 7.374 |
| 24 H | 6.5 | 7.6 | 6.8 | 38 | 0.6 | 34.9 | 7.392 |
| 28 H | 6.9 | 7.7 | 7.6 | 36 | 0.8 | 27.6 | 7.379 |
| 32 H | 5.3 | 4.9 | 4.1 | 36 | 0.8 | 37.8 | 7.380 |
| 36 H | 7.7 | 6.4 | 5.4 | 46 | 0.8 | 30.4 | 7.362 |
| 40 H | 5.8 | 5.2 | 5.2 | 40 | 0.8 | 31.0 | 7.343 |
| 44 H | 5.8 | 5.2 | 4.5 | 39 | 0.7 | 38.4 | 7.348 |
| 48 H | 5.7 | 5 | 4.1 | 40 | 0.6 | 36.2 | 7.342 |
| 52 H | 6.8 | 7.5 | 7.3 | 37 | 0.8 | 34.4 | 7.340 |
| 56 H | 8.8 | 8.2 | 7.9 | 35 | 0.6 | 44.3 | 7.317 |
| 60 H | 8.1 | 8.3 | 9.2 | 37 | 0.8 | 33.1 | 7.343 |
| 64 H | 6.5 | 5.7 | 5.1 | 40 | 0.9 | 28.9 | 7.339 |
| 68 H | 8.2 | 9.8 | 9 | 35 | 0.7 | 38.2 | 7.339 |
| 72 H | 4.9 | 6.4 | 5.5 | 35 | 0.7 | 37.9 | 7.347 |

Glucose concentration in mmol/L performed every fourth hour with CGMS, POC glucometer and blood gas analyses. Physiological status acquired from the blood gas analyser measurements (HCT, lactate, O_2_, CO_2_ and pH)

**Case 2**

**Table xx. Case 2. Results of glucose measurements and physiological status.**

| Time (Hours) | CGMS  (mmol/L) | Blood gas (mmol/L) | POC  (mmol/L) | Hematocrit (%) | Lactate (mmol/L) | pO_2_  _(_mmHg) | pH |
| --- | --- | --- | --- | --- | --- | --- | --- |
| 0 H | 13.2 | 12.3 | 13.2 | 35 | 1.1 | 37.4 | 7.373 |
| 4 H | 7.9 | 6.6 | 6.1 | 38 | 0.6 | 39.0 | 7.342 |
| 8 H | 7 | 6.6 | 6.1 | 40 | 0.7 | 43.2 | 7.321 |
| 12 H | 6.4 | 6.5 | 6.2 | 31 | 0.5 | 29.4 | 7.308 |
| 16 H | 7.2 | 6.9 | 6.8 | 31 | 0.5 | 38.5 | 7.308 |
| 20 H | 6.3 | 5.6 | 5.2 | 33 | 0.5 | 29.9 | 7.299 |
| 24 H | 8.1 | 7 | 6.7 | 30 | 0.5 | 35.0 | 7.295 |
| 28 H | 6.7 | 6.6 | 6.4 | 31 | 0.6 | 32.8 | 7.309 |
| 32 H | 7.4 | 7.3 | 7.3 | 32 | 0.6 | 34.2 | 7.315 |

Glucose concentration in mmol/L performed every fourth hour with CGMS, POC glucometer and blood gas analyses. Physiological status acquired from the blood gas analyser measurements (HCT, lactate, O_2_, CO_2_ and pH)

Case 3

| Time (Hours) | CGMS  (mmol/L) | Blood gas  (mmol/L) | POC  (mmol/L) | Hematocrit (%) | Lactate (mmol/L) | pO_2_  (mmHg) | pH |
| --- | --- | --- | --- | --- | --- | --- | --- |
| 0 H | 11.8 | 10.8 | 11.8 | 39 | 1.9 | 34.2 | 7.404 |
| 4 H | 10 | 9.3 | 9.7 | 40 | 1.7 | 35.8 | 7.409 |
| 8 H | 7 | 6.6 | 6.6 | 40 | 1.3 | 32.4 | 7.391 |
| 12 H | 5 | 5.5 | 5.3 | 43 | 1.4 | 33.33 | 7.381 |
| 16 H | 4.8 | 8 | 8.3 | 34 | 1.6 | 34.4 | 7.377 |
| 20 H | 10.2 | 11.6 | 12 | 35 | 1.2 | 34.2 | 7.355 |
| 24 H | 9.9 | 7.8 | 7.2 | 40 | 1.4 | 32.0 | 7.368 |
| 28 H | 11.7 | 8.4 | 9.2 | 28 | 1.7 | 33.4 | 7.331 |
| 32 H | 10.8 | 11.4 | 12.6 | 37 | 2.2 | 38.2 | 7.325 |
| 36 H | 8.7 | 8.1 | 8.6 | 33 | 3.2 | 36.9 | 7.325 |
| 40 H | 7.8 | 7.6 | 7.9 | 34 | 2.6 | 25.4 | 7.337 |

Glucose concentration in mmol/L performed every fourth hour with CGMS, POC glucometer and blood gas analyses. Physiological status acquired from the blood gas analyser measurements (HCT, lactate, O_2_, CO_2_ and pH)

Case 4

| Time (Hours) | CGMS  (mmol/L) | Blood gas  (mmol/L) | POC  (mmol/L) | Hematocrit (%) | Lactate  (mmol/L) | pO_2_  (mmHg) | pH |
| --- | --- | --- | --- | --- | --- | --- | --- |
| 0 H | 6.9 | 6.2 | 6.9 | 18 | 4.7 | 54.2 | 7.244 |
| 4 H | 5.8 | 5.6 | 5.9 | 22 | 4.7 | 56.3 | 7.273 |
| 8 H | 5.9 | 5.5 | 5.9 | 24 | 4.0 | 44.0 | 7.260 |
| 12 H | 5.9 | 6.1 | 6.5 | 25 | 3.6 | 31.9 | 7.293 |
| 16 H | 5.9 | 5.8 | 6.5 | 28 | 3.0 | 30. | 7.304 |
| 20 H | 6.3 | 5.9 | 5.9 | 28 | 2.9 | 30.8 | 7.310 |
| 24 H | 5.6 | 4.6 | 4.7 | 29 | 2.6 | 34.6 | 7.263 |
| 28 H | 6.3 | 6.6 | 6.4 | 34 | 2.8 | 23.2 | 7.315 |
| 32 H | 6.3 | 6.8 | 7.7 | 33 | 3.4 | 28.1 | 7.284 |
| 36 H | 6.4 | 6.2 | 6.5 | 28 | 3.1 | 28.8 | 7.286 |
| 40 H | 5.1 | 7.8 | 8.3 | 27 | 8.1 | 20.7 | 7.244 |

Glucose concentration in mmol/L performed every fourth hour with CGMS, POC glucometer and blood gas analyses. Physiological status acquired from the blood gas analyser measurements (HCT, lactate, O_2_, CO_2_ and pH)

Case 5

| Time (Hours) | CGMS (mmol/L) | Blood gas (mmol/L) | POC (mmol/L) | Haematocrit  (%) | Lactate  (mmol/L) | pO_2_  (mmHg) | pH |
| --- | --- | --- | --- | --- | --- | --- | --- |
| 0 H | 7.2 | 6.8 | 7.2 | 34 | 1.5 | 33 | 7.387 |

Case 6

| Time (Hours) | CGMS (mmol/L) | Blood gas (mmol/L) | POC (mmol/L) | Haematocrit (%) | Lactate (mmol/L) | pO_2_  (mmHg) | pH |
| --- | --- | --- | --- | --- | --- | --- | --- |
| 0 H | 8.3 | 8.3 | 8.3 | 33 | 0.6 | 44.8 | 7.383 |

Case 8

| Time (Hours) | CGMS  (mmol/L) | Blood gas  (mmol/L) | POC  (mmol/L) | Hematocrit  (%) | Lactate (mmol/L) | pO_2_  (mmHg) | pH |
| --- | --- | --- | --- | --- | --- | --- | --- |
| 0 H | 11.6 | 12.6 | 11.6 | 47 | 4.9 | 37.5 | 7.300 |
| 4 H | 10 | 10.5 | 9.6 | 48 | 2.4 | 33.4 | 7.378 |
| 8 H | 10.7 | 11.7 | 9.9 | 50 | 1.9 | 40.8 | 7.419 |
| 12 H | 6.8 | 6.4 | 4.9 | 50 | 1.4 | 43.3 | 7.416 |
| 16 H | 6.3 | 6.1 | 4.5 | 52 | 1.5 | 43.5 | 7.400 |
| 20 H | 4.8 | 7 | 5.9 | 45 | 1.9 | 37.6 | 7.385 |
| 24 H | 5.9 | 7.8 | 7.3 | 43 | 2.1 | 39.0 | 7.389 |
| 28 H | 4.3 | 5.8 | 4.7 | 43 | 2.9 | 33.0 | 7.379 |
| 32 H | 6.8 | 8 | 6.8 | 47 | 6.0 | 40.6 | 7.340 |
| 36 H | 7.5 | 9.3 | 8.5 | 54 | 7.8 | 33.8 | 7.304 |
| 40 H | 11.7 | 14.5 | 14.6 | 42 | 9.5 | 40.9 | 7.291 |
| 44 H | 9.1 | 11.1 | 10.7 | 44 | 10.7 | 42.2 | 7.222 |
| 48 H | 8 | 9.6 | 9.3 | 43 | 14.1 | 33.5 | 7.178 |
| 52 H | 9.1 | 7.9 | 8 | 44 | 14.7 | 36.8 | 7.132 |

Case 9

| Time (Hours) | CGMS  (mmol/L) | Blood gas  (mmol/L) | POC  (mmol/L) | Hematocrit (%) | Lactate (mmol/L) | pO_2_  (mmHg) | pH |
| --- | --- | --- | --- | --- | --- | --- | --- |
| 0 H | 8.1 | 8.4 | 8.1 | 37 | 1.1 | 32.1 | 7.402 |

Case 10

| Time (Hours) | CGMS  (mmol/L) | Blood gas  (mmol/L) | POC  (mmol/L) | Hematocrit  (%) | Lactate (mmol/L) | pO_2_  (mmHg) | pH |
| --- | --- | --- | --- | --- | --- | --- | --- |
| 0 H | 7.1 | 7.6 | 7.1 | 35 | 0.6 | 28.8 | 7.335 |
| 4 H | 4.8 | 8.5 | 8.2 | 30 | 0.6 | 32.5 | 7.321 |
| 8 H | 5.8 | 7.7 | 7.7 | 29 | 0.5 | 25.0 | 7.301 |
| 12 H | 5.4 | 5.8 | 4.7 | 34 | 0.6 | 37.6 | 7.350 |
| 16 H | 6.1 | 7.8 | 8 | 31 | 0.5 | 30.7 | 7.338 |
| 20 H | 7.7 | 8 | 8.1 | 32 | 0.6 | 29.3 | 7.336 |

**Table XX. Sensor and time overview in adult horse cases**

| Adult horse cases | Number of CGMS sensors used | Hours from placement of first sensor until CGMS monitoring starts (including initialization period of two hours ) | Total hours of glucose CGMS monitoring (hours after initialization period of two hours) |
| --- | --- | --- | --- |
| 1 | 5 | 5 | 71.5 |
| 2 | 3 | 5 | 32.5 |
| 3 | 1 | 2.25 | 39.83 |
| 6 | 3 | 6.5 | 1.58 |
| 8 | 1 | 2.08 | 48.92 |
| 10 | 1 | 2.2 | 19.67 |
| Mean ±SD | **2.33 ±1.63** | **3.84 ±1.9** | **35.67 ± 24.1** |

**Table XX. Sensor and time overview in foal cases**

| Foal cases | Number of CGMS sensors used | Hours from placement of first sensor until CGMS monitoring starts (including initialization period of two hours ) | Total hours of glucose CGMS monitoring (hours after initialization period of two hours) |
| --- | --- | --- | --- |
| 4 | 1 | 2.25 | 40.17 |
| 5 | 5 | 3.75 | 0.83 |
| 7 | 4 | 0 | 0 |
| 9 | 3 | 5.25 | 1.75 |
| Mean | **3.25 ±1.71** | **2.81 ±2.24** | **10.69 ±19.67** |
