# Supplementary material for: Blood glucose and subcutaneous continuous glucose monitoring in critically ill horses: A pilot study
Source: PLoS One. 2021 Feb 24;16(2):e0247561. doi: 10.1371/journal.pone.0247561 (PMC7904136; doi:10.1371/journal.pone.0247561)
Supplement: Attachment — Submitted filename: Revision PONE-D-20-35136.docx [file pone.0247561.s008.docx]

The authors’ aim was to verify the feasibility of use a new devise to dose interstitial glucose concentration and the correlation between interstitial and blood glucose (evaluated by PC and blood gas analyzer). The paper is interesting and well structured, but it needs some corrections to be suitable for publication.

Lines 79-84: SIRS in foals: Please you need to use the SIRS score proposed by Roy et al 2017 for adult horse and the one by Wong and Wilkins in 2015 (and 2018 for sepsis) for foals. Please correct and verify if your population can still be included as SIRS positive horses and foals.

I see the data have been collected in 2016, thus maybe you can add tìsome explanation about why you have used a not recent paper for the adult SIRS parameters, but the paper by Wong and Wilkins has been published in the 2015, thus you need to use these parameters for foals’ inclusion in SIRS positive group.

Line 89: was the catheter used only for blood samples? Please add information.

Line 95: what do you mean for “validated”? Please add information.

Line 98: PCV has been evaluated as microhematocrit? Please clarify. After you never discuss this parameter, and this is not relevant for the SIRS inclusion/exclusion. So why did you report this parameter?

Line 113: are the cut-off supported by literature or the cut-off have been chosen by you? Please clarify.

Line 128-129: compared applying what? Bland altman? I think yes (see lines 131-133). If so, in this case you need to better rephrase the sentence in order to have a more understandable and “easy to read” sentence.

Line 131-132: why? This is not one of the aims of the study. In my opinion it should be erased, or you need to change the aim and the title.

Line 136: please add literature for glucose ranges.

Lines 138-139: you say what you did but not haw statistically. Please add.

Table 1: please change “range” with minimum and maximum values. Please add “total number of sensors used until….” in the first row.

Lines 172-173: as my previous comment, this is not one of the aims of the study. In my opinion it should be erased, or you need to change the aim and the title.

Line 174-175: this sentence needs to be moved to mat and met section.

Line 178: fair correlation: both for POC and BGA? You need to specify and discuss both.

Table 2 and Fig. 2. Please erase the comparison between the POC and the blood gas because this is not the aim you stated. The aim was to compare the CGM with BGA or the POC, thus the comparison must be done between CGM vs BGA or POC, not between BGA and POC.

Lines 204-205: again, comparison between BGA and POC is not an aim of your study.

Lines 234 and 247: as previously said.

Lines 261: differs

Line 273: add human patient.

Line 297: in the discussion you said the use of the devise is very difficult in foals, thus this consideration seems to be in contrast with your previous idea. Please clarify or change or erase.

Literature

Corley is 2005 and not 2015.
